# Parent of Origin Effects on Family Communication of Risk in *BRCA*+ Women: A Qualitative Investigation of Human Factors in Cascade Screening

**DOI:** 10.3390/cancers12082316

**Published:** 2020-08-17

**Authors:** Andrew A. Dwyer, Sharlene Hesse-Biber, Bailey Flynn, Sienna Remick

**Affiliations:** 1 Boston College, William F. Connell School of Nursing, Chestnut Hill, MA 02467, USA; andrew.dwyer@bc.edu; 2 Munn Center for Nursing Research, Massachusetts General Hospital, Boston, MA 02114, USA; 3 Boston College, Department of Sociology, Chestnut Hill, MA 02467, USA; baileyflynn2024@u.northwestern.edu (B.F.); remicknsisi@gmail.com (S.R.)

**Keywords:** BRCA mutation, cascade genetic testing, coping cancer, family communication, genetic testing, theory of planned behavior

## Abstract

Pathogenic germline variants in Breast Cancer 1/2 (*BRCA*) genes confer increased cancer risk. Understanding *BRCA* status/risk can enable family cascade screening and improve cancer outcomes. However, more than half of the families do not communicate family cancer history/*BRCA* status, and cancer outcomes differ according to parent of origin (i.e., maternally vs. paternally inherited pathogenic variant). We aimed to explore communication patterns around family cancer history/*BRCA* risk according to parent of origin. We analyzed qualitative interviews (*n* = 97) using template analysis and employed the Theory of Planned Behavior (TPB) to identify interventions to improve communication. Interviews revealed sub-codes of ‘male stoicism and ‘paternal guilt’ that impede family communication (template code: gender scripting). Conversely, ‘fatherly protection’ and ‘female camaraderie’ promote communication of risk. The template code ‘dysfunctional family communication’ was contextualized by several sub-codes (‘harmful negligence’, ‘intra-family ignorance’ and ‘active withdrawal of support’) emerging from interview data. Sub-codes ‘medical misconceptions’ and ‘medical minimizing’ deepened our understanding of the template code ‘medical biases’. Importantly, sub-codes of ‘informed physicians’ and ‘trust in healthcare’ mitigated bias. Mapping findings to the TPB identified variables to tailor interventions aimed at enhancing family communication of risk and promoting cascade screening. In conclusion, these data provide empirical evidence of the human factors impeding communication of family *BRCA* risk. Tailored, theory-informed interventions merit consideration for overcoming blocked communication and improving cascade screening uptake.

## 1. Introduction

Breast cancer is the most common female cancer diagnosis and approximately 2.1 million women were newly diagnosed with breast cancer in 2018 [1]. Approximately 10% of all breast cancers are inherited and associated with family history [2]. Notably, women harboring pathogenic variants in *BRCA1* or *BRCA2* have increased risk for developing breast cancer (72% and 69% respectively by age 80) [3]. Thus, detection of *BRCA* pathogenic variants has significant relevance for medical management as earlier detection and risk reducing strategies (i.e., hormonal treatment, risk-reducing surgery) can improve outcomes. The United States Preventive Services Task Force has recently updated evidence-based recommendations for risk assessment, genetic counseling and genetic testing for *BRCA* [4]. Importantly, once a pathogenic *BRCA* variant has been identified, screening can be extended to include other blood relatives who may carry the pathogenic variant (termed cascade screening). The U.S. Centers for Disease Control and Prevention (CDC) recognizes hereditary breast and ovarian cancer (HBOC) syndrome as a Tier 1 condition meaning that evidence-based guidelines and recommendations support a significant potential for positive public health impact. Accordingly, the National Cancer Institute, the Cancer Moonshot Blue Ribbon Panel and the Genomics Population Health Action Collaborative have called for greater implementation of cascade screening for breast cancer [5].

Pathogenic *BRCA* variants may be inherited from either parent. Interestingly, paternally inherited pathogenic *BRCA* variants have been associated with worse outcomes—including earlier cancer onset and more aggressive disease [6,7,8]. However, paternally inherited pathogenic *BRCA* variants are neither more penetrant nor more virulent than maternally inherited pathogenic variants, and parent of origin effects disappear when controlling for referral bias [9,10]. Data suggest that paternal family history is minimized and often neglected [8,11,12,13]. Thus, offspring harboring paternally inherited pathogenic *BRCA* variants may underestimate their cancer risk. Women with a paternally inherited pathogenic *BRCA* variant are less likely to receive genetic counseling, less likely to have genetic testing prior to cancer diagnosis, and are more likely to be receive a cancer diagnosis [14]. Conversely, women with maternally inherited pathogenic variants are almost three times more likely to have a proactive response towards genetic testing (i.e., testing before receiving a cancer diagnosis) [14].

Two important human factors underlie effective cascade screening. First, individuals must perceive and understand genetic risk. Second, information needs to be communicated throughout the family to at-risk relatives. Perception of genetic risk is multifactorial and is influenced by a family history of cancer, fertility plans and communication with healthcare professionals [15,16]. Recent studies indicate that less than half of families fully communicate risk to at-risk relatives [17] and only about one-half of relatives have cascade screening [18,19,20,21]. As indicated in a recent scoping review, best practices for optimizing cascade screening do not exist and very few studies have rigorously tested interventions to improve the process [5]. Authors identified suboptimal family communication as a major barrier to more widespread implementation [5]. Previous work has hypothesized that ineffective communication arising from stigma and shame may contribute to referral bias for *BRCA* [9]. Similarly, themes of “blocked” family communication have been proposed as potential explanations for divergent health and wellbeing outcomes [14]. Given the complexity of family communication, recent work suggests theoretical frameworks may be useful for dissecting components to inform interventions [16]. This is particularly relevant because effective family communication of genetic risk has been identified as a modifiable factor in facilitating cascade screening [18]. There is substantial evidence that theory-informed interventions are more effective than purely empirical or pragmatic approaches [22]. Accordingly, the United Kingdom Medical Research Council recommends integrating relevant theories/frameworks when developing complex interventions [23]. One theory, the Theory of Planned Behavior (TPB) [24], has been effectively used in the context of breast cancer to describe a wide range of cancer-related behaviors including screening/mammography [25], risk-reducing surgeries [26,27], lymphedema risk reduction activities [28], adherence to adjuvant endocrine therapies [29], as well as contacting potentially at-risk relatives [30]. Similarly, the TPB has been employed in the field of genetic counseling [31] to better understand and predict behaviors around prenatal genetic testing [32] and expanded carrier screening [33].

A better understanding of parent of origin barriers/facilitators of family communication of risk is important for harnessing the full potential of cascade screening and improving *BRCA* cascade screening outcomes. Elucidating the communication process in families harboring pathogenic *BRCA* variants may help promote theory-informed approaches [34] to overcoming some of the human-related barriers to cascade screening. This study aims to examine communication patterns in families harboring pathogenic *BRCA* variants. In particular, we analyze qualitative data from 97 semi-structured interviews to explore communication regarding family history of cancer in relation to parent of origin. We use themes drawn from the literature to produce a coding template followed by a grounded theory approach to identify additional sub-codes emerging from the interview data (i.e., thematic template analysis, see Materials and Methods). We employed the TPB [24] as a guiding framework for understanding family communication of genetic risk. Additionally, we identify targets at the individual, family and health system level for facilitating intrafamilial communication and enabling cascade screening.

## 2. Results

A total of 97 *BRCA*+ women were interviewed for this study. Nearly two-thirds of women (62/97, 64%) had inherited the pathogenic variant from their mother, while 35/97 (36%) had a paternally inherited pathogenic *BRCA* variant. Demographic characteristics did not differ by parent of origin (Table 1). Participants were overwhelmingly white/Caucasian who were well educated (at least some college) and self-identified as being middle class. The rates of *BRCA* testing within specified age brackets did not differ according to paternal vs. maternal inheritance (18–25 yrs 4/32 vs. 13/57 *p* = 0.24; 26–30 yrs: 6/32 vs. 13/57 *p* = 0.65; 31–35 yrs: 4/32 vs. 8/57 *p* = 0.84; 36–40 yrs: 7/32 vs. 7/57 *p* = 0.23; 41–50 yrs: 8/32 vs. 11/57 *p* = 0.53; 51–60 yrs: 2/32 vs. 4/57 *p* = 0.89; 61–70 yrs: 1/32 vs. 1/57 *p* = 0.68). These findings differ from prior reports [6,7,8,14], demonstrating that women harboring maternally inherited *BRCA* variants have genetic testing earlier than their paternally inherited counterparts do. The discordant findings are likely due to smaller sample size of the present qualitative investigation compared to previous quantitative studies. However, women with paternally inherited pathogenic *BRCA* variants were more likely to have been diagnosed with cancer compared to maternally inherited counterparts (13/34 vs. 9/62, χ^2^ = 6.99, *p* = 0.008). We then sought to examine these differences using qualitative inquiry. There are three levels providing structure for the qualitative analysis. The broadest category includes “template themes” that serve as central organizing concepts for understanding the research question. Iterative qualitative analysis (termed coding) identifies emergent “sub-codes” that are relevant concepts giving meaning to the template theme. Lastly, “dimensions” are elements that provide context for the sub-codes.

### 2.1. Template Theme 1: ‘Gender Scripting’

**Figure 1:**
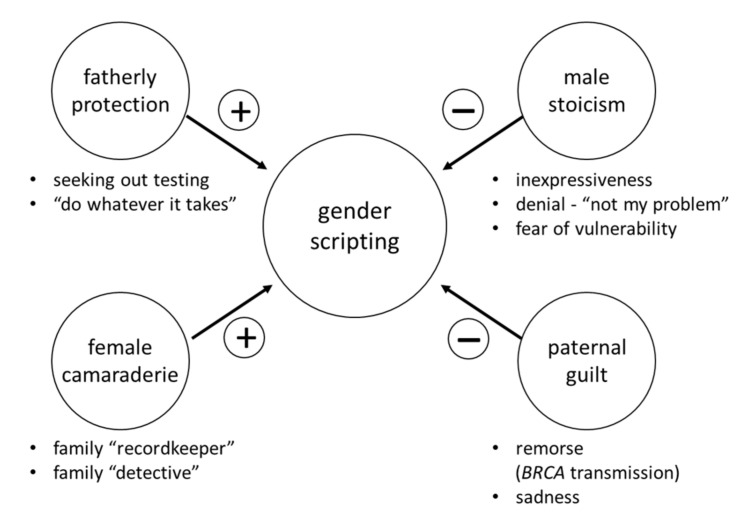
Sub-codes mapping to the template theme ‘gender scripting’ in paternally inherited pathogenic *BRCA* variants. Analyzing interviews (*n* = 35) with women harboring paternally inherited pathogenic *BRCA* variants identified four sub-codes (small circles) and related dimensions (bulleted) that are linked to the template theme of ‘gender scripting’. Two sub-codes facilitated intrafamilial communication of risk (+) while two contributed to blocked communication (−).

Women harboring paternally inherited pathogenic *BRCA* variants shared stories highlighting the template theme of ‘gender scripting’ (i.e., gender-specific roles and activities). Iterative coding of interview data identified sub-codes providing further insight into how ‘gender scripting’ either promoted or inhibited communication of risk within families and ‘paternal guilt’ respectively (Figure 1).

For example, the sub-code ‘fatherly protection’ included dimensions of actively ‘seeking out genetic testing’ to inform the family and ‘doing whatever it takes’ to be supportive of their daughter (Table 2). The sub-code ‘female camaraderie’ emerged from stories of mothers and aunts being the “keepers” of family history. Such women assumed a “detective” role to identify risk and subsequently took action and shared information with at-risk relatives (Table 2). Conversely, traditional male roles could also inhibit family communication of risk. The sub-code ‘male stoicism’ included daughter-identified dimensions including ‘inexpressiveness’, ‘denial’ (i.e., “not my problem”) and ‘fear of being vulnerable’. Interestingly, some interviewees noted that female family members taking on the role of “family detective” and “*BRCA* informant” (i.e., sub-code ‘female camaraderie’) helped surmount blocked communication resulting from male stoicism. The ‘Paternal guilt’ sub-code emerged as the negative counterpoint to the ‘fatherly protection’ sub-code. ‘Paternal guilt’ was marked by dimensions of ‘sadness’ and ‘remorse’ for passing the pathogenic *BRCA* variant to the daughter. Women with maternally inherited pathogenic *BRCA* variant also exhibited distinct ‘gender scripts’ around communication. Specifically, mothers frequently acted as a “guide” for their daughters in relation to *BRCA* risk. Indeed, mothers often took on the “*BRCA* informant” role. This active role in communicating risk within families was the counterpoint to general reluctance to discuss *BRCA* risk among men in paternally inherited families.

### 2.2. Template Theme 2: ‘Family Dynamics’

**Figure 2:**
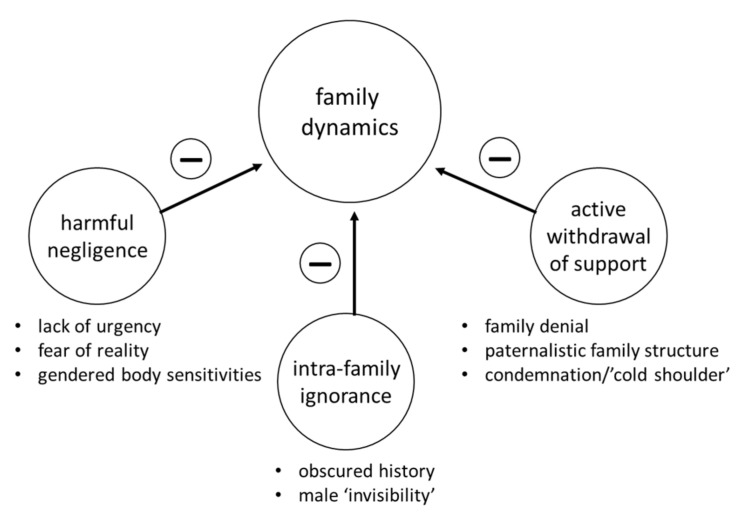
Sub-codes mapping to the template theme ‘family dynamics’. Analyzing the 97 interviews revealed three sub-codes (small circles) negatively affecting (−) family dynamics and impeding effective communication. Sub-code dimensions are depicted by bullets.

Interviews revealed three main sub-codes negatively affecting the template theme of ‘family dynamics’ (Figure 2). Sub-codes emerged from both maternally and paternally inherited *BRCA* families. The sub-code ‘harmful negligence’ included dimensions relating to ‘lack of urgency’ and/or ‘fear of reality’ that *BRCA* risk existed in the family (Table 3). Women feeling uncomfortable discussing breasts/ovaries/reproduction with male relatives (i.e., dimension: ‘gendered body sensitivities’) also emerged as a barrier to communication. Another emergent finding relating to ‘family dynamics’ was the sub-code ‘intra-family ignorance’ that prevented the recognition of clinical red flags raising the suspicion of hereditary cancer risk. Familial ignorance included dimensions of ‘obscured family history’—wherein families were unaware or did not recognize their family cancer history and risk. Limited genetic literacy was a barrier as family members did not understand that men were able to inherit pathogenic *BRCA* variants and pass them on to their offspring, effectively rendering males invisible when identifying risk (dimension: ‘male invisibility’). While ‘harmful negligence’ and ‘intra-family ignorance’ sub-codes are unintentional, the third emergent sub-code reflected deliberate action. ‘Active withdrawal of support’ represents a conscious decision to pull away from a *BRCA*+ family member. Associated dimensions include avoidant family coping (i.e., ‘family denial’), a dominant father figure (‘paternalistic family structure’) and refusal of support for the family member affected by *BRCA* (i.e., ‘condemnation/cold shoulder’)

Notably, we observed the dimension of ‘family denial’ was much more prominent in interviews of women harboring a paternally inherited pathogenic *BRCA* variant. Through the eyes of their daughters, we see that fathers often kept familial risk to themselves. Women harboring paternally inherited pathogenic variants frequently described their fathers as reluctant to discuss cancer/*BRCA* and often chose non-disclosure over facing the perceived stigma of being a *BRCA*+ man. Such experiences cultivated a culture of silence that delayed genetic testing and was potentially harmful to all at-risk relatives who could benefit from cascade screening (Table 3). Women considered their fathers’ ‘active withdrawal of support’ and ‘denial’ as a reaction to *BRCA* stigma and perceived indictment of paternal culpability. Many interviewees also considered that their *BRCA*+ status and potential mortality was perceived as a threat to family stability. Emergent themes relating to ‘family dynamics’ highlight both unintentional and intentional barriers to effective intrafamilial communication. Such barriers enable familial cancer risk to go undetected for generations and prevent cascade screening. When *BRCA* risk remained unknown in families, stories of secrecy, silence, emotional distance and discomfort discussing medical history were often noted in interviews (Table 3). Women also cited geographic distance, socioeconomic differences and disparate lifestyles as reasons contributing to lack of communication regarding *BRCA* risk. These observations are consistent with previously reported barriers to cascade screening [5].

### 2.3. Template Theme 3: ‘Medical Biases’

**Figure 3:**
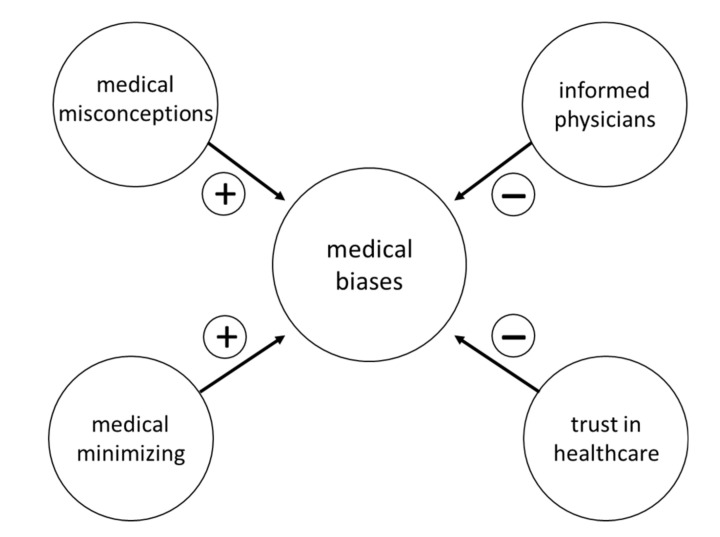
Sub-codes mapping to the template theme ‘medical biases’. Analyzing the 97 interviews identified four sub-codes relating to biases. Two codes contributed to healthcare provider biases (+) and two were mitigating factors (−).

Interviews revealed sub-codes related to ingrained misconceptions and implicit biases among healthcare professionals regarding gender that facilitate/impede understanding risk imparted by pathogenic *BRCA* variants. Both ‘medical misconceptions’ and ‘medical minimizing’ emerged as sub-codes undermining accurate assessment of *BRCA* risk. In contrast, other interviews identified factors promoting accurate assessment of familial cancer (*BRCA*) risk. Women appreciated healthcare professionals who understood that *BRCA* can be passed via maternal or paternal inheritance (i.e., sub-code: ‘informed providers’). Another mitigating factor of ‘medical biases’ was the sub-code of ‘trust in healthcare’ (i.e., patient-centered care, healthcare professionals who placed patient concerns at the center of discussions and decisions) (Figure 3). Notably, quotes drawn from interviews point to a matrix of gender role influences on how some healthcare professionals assess *BRCA* risk assessment and how some families communicate *BRCA* risk (Table 1 and Table 4).

### 2.4. Mapping Template Themes and Sub-Codes to the Theory of Planned Behavior (TPB)

**Figure 4:**
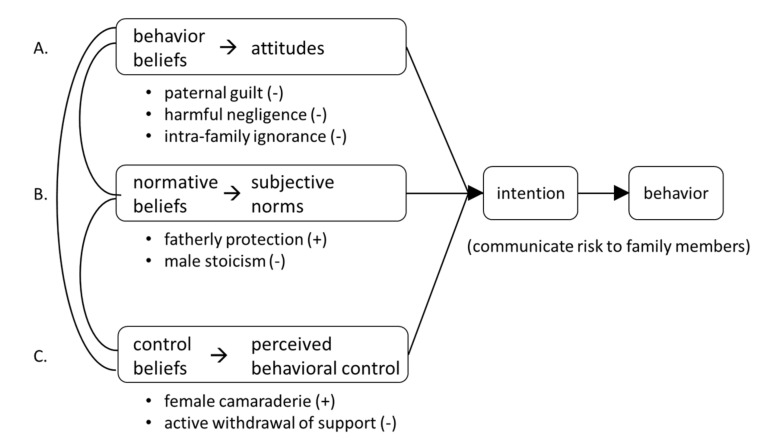
Interview sub-codes mapped to the Theory of Planned Behavior (TPB). (**A**) The TPB posits that beliefs shape attitudes that influence intention which precedes behavior; (**B**) social and cultural beliefs shape perceived social pressures creating subjective norms that influence intention; (**C**) control beliefs shape perceived behavioral control (i.e., self-efficacy, perceptions of one’s ability to perform the behavior). Sub-codes (bullets) emerging from the two family-related template themes (‘gender scripting’ and ‘family dynamics’) were mapped to the TPB to elucidate the matrix of patient and family-related factors that either promote (+) or inhibit (−) communication of risk and subsequent cascade screening.

Cascade screening hinges on intrafamilial communication of *BRCA* risk. However, family communication is complex and dynamic. We used the TPB [24] to better understand the revelation of *BRCA* family risk as a key element for cascade screening. Sub-codes were mapped onto the TPB to better understand the interplay of factors influencing family communication around *BRCA* risk (Figure 4). Briefly, the TPB posits that experiences and beliefs shape attitudes (i.e., good/bad) towards a particular behavior (i.e., family communication of risk). We considered three sub-codes relating to decreased/ineffective family communication of risk (‘paternal guilt’, ‘harmful negligence’, ‘intra-family ignorance’) as relating to TPB attitudinal factors. In the TPB, subjective norms relate to social/family norms (i.e., whether most people approve or disapprove). Interestingly, sub-codes under this category relate to traditional male roles yet represent opposite sides of the coin so to speak. The sub-code ‘male stoicism’ (i.e., being a strong male) was associated with limited family communication of risk. Conversely, ‘fatherly protection’ (safeguarding the family) fostered communication of family risk. The TPB posits perceived behavioral control relates to an individual’s sense of agency and self-efficacy for performing a particular behavior. Gender roles represented both inhibiting and promoting factors for family communication. ‘Female camaraderie’ helped promote *BRCA* awareness in families. Indeed, female family members often felt “empowered” and “obliged” to communicate risk. Alternatively, family members who were unable to cope with family risk and/or *BRCA* stigma (i.e., low self-efficacy for communication) often reacted with ‘active withdrawal of support’.

To identify targets for interventions aimed at enhancing family communication of risk and promoting cascade screening, we combined the TPB mapping exercise with sub-codes relating to the healthcare system (template theme: ‘medical biases’). This ecological approach identified individual, family and institutional targets for interventions [35]. Proposed interventions (Table 5) targeting the individual include employing principles of behavioral economics and motivational interviewing.

## 3. Discussion

Herein we present data from 97 qualitative interviews with *BRCA*+ women to better understand family communication around *BRCA* cancer risk that precedes cascade screening. Previous studies have demonstrated differences between family members in reporting maternal and paternal history of cancer respectively [11,12,16]. Additionally, we have previously shown differences in approaches to risk management and psychosocial burden according to parent of origin [14]. Indeed, women with maternally inherited pathogenic *BRCA* variants more frequently report open family communication, being aware/informed of risk, feeling more empowered and are more likely to exhibit proactive risk management as well as better health and psychosocial outcomes [14]. Using template analysis using themes drawn from existing literature, we sought to consider sex as a variable to parse the role of communication in the divergent parent of origin outcomes. A comprehensive literature review identified three template themes (gender scripting, family dynamics, medical biases) that guided our examination of *BRCA* parent of origin effects on intrafamilial communication. The emergent sub-codes (and dimensions) in the present study provide a broad picture of facilitators and barriers to discussing *BRCA* risk in families with important ramifications for cascade screening. Notably, we report a matrix of gender role influences that exist within families and among healthcare professionals.

The literature consistently identifies several universal barriers to effective family communication of risk and cascade screening including poor knowledge/understanding of genetics and heritability, limited communication skills, financial barriers (i.e., cost, insurance coverage), anxiety/depression in probands and/or relatives and geographic barriers to accessing genetic services [5]. Studies have noted the uptake of cascade screening to be lower in male relatives compared to females. However, the reasons for lower uptake of testing among males have yet to be elucidated. This study presents qualitative empirical data providing insights into the human factors and potential mechanisms (including parent of origin) that help explain the observed sexual discordance in cascade screening. Qualitative inquiry is recognized as an essential component of developing complex interventions [22,23], and the present findings can inform tailored interventions for families. Best practices for developing and testing complex interventions include the incorporation of theories/frameworks to guide the process [22,23]. Thus, mapping our findings onto the Theory of Planned Behavior (TPB) contributes to advancing novel, tailored family interventions promoting cancer predisposition cascade genetic testing for *BRCA*. This theoretical contribution towards developing complex interventions promoting cascade screening is further supported by the demonstrated utility of the TPB in breast cancer and genetic counseling [25,26,27,28,29,30,31,32,33].

Gender scripting is a major theme contributing to parent of origin differences in medical and psychosocial outcomes [14]. Traditional western gender roles consider males as strong, assertive, independent providers and protectors, while females have greater emotional sensitivity, are more sociable and have a greater concern for others [36]. Such gender scripts can have positive benefits for promoting family communication such as ‘female camaraderie’—a relative who takes on the role of ‘family record-keeper’ and ‘detective’ to emerge as the family “BRCA informant”. Similarly, men who identify with the protector role (sub-code: ‘fatherly protection’) sought out testing and were dedicated to doing “whatever it takes” to help their daughter. Interviews revealed that gender roles and implicit biases often impede open discussion of *BRCA* risk within families. Conversely, shadow sides of male roles took the form of ‘parental guilt’ for transmitting the pathogenic variant (i.e., not being a good protector) and ‘male stoicism’ (e.g., dimensions of ‘denial’ and ‘inexpressiveness’). Gender scripts also contributed to the sub-code of ‘intra-family ignorance’. In particular, misperceptions that *BRCA* status relates only to breast cancer (i.e., a “woman’s disease”) contributed to ‘stigma’ and the dimension of ‘male invisibility’. Male history was often invisible, thus red flags were not recognized in family history and risk was not communicated within families. Similarly, gender roles created barriers for male-female communication. Specifically, individuals noted family member discomfort with talking about their body with relatives of the opposite sex (dimension: ‘gendered body sensitivities’). Emotional responses to *BRCA* cancer risk contributed to dysfunctional family dynamics demonstrated by reports of ‘active withdrawal of support’ for the *BRCA* affected family member. Daughters interpreted male emotional retreat as stemming from denial and fear of parental culpability for passing the pathogenic *BRCA* variant on to them. Notably, gendered scripting and implicit biases were not limited to family members. Some healthcare professionals hold ‘medical misconceptions’ (e.g., *BRCA* can only be inherited from the maternal line) that contribute to ‘medical minimizing’ (brushing off red flags from family history) (Figure 3, Table 4). Both misconceptions and minimizing can result in delays in seeking care. Notably, delayed care seeking is particularly evident among women harboring paternally inherited pathogenic *BRCA* variants [14]. Interviews detailed the way gender scripting played out in families and points to human factors contributing to the poorer outcomes observed in women who inherit pathogenic *BRCA* variants from their fathers [14]. Women noted that gender scripting negatively influenced intrafamilial communication. Indeed, women with a paternally inherited pathogenic *BRCA* variant described communication as “blocked” and felt “blindsided” by *BRCA* and cancer [14].

Hereditary cancer resulting from pathogenic *BRCA* variants has implications for the entire family. Accordingly, open family communication of *BRCA* risk cancer can be a catalyst for cascade screening and risk reducing strategies, including aggressive surveillance, hormone treatment and/or surgery. Open family communication has been associated with decreased distress [37]. However, families have variable responses to *BRCA* status ranging from complete openness to limited disclosure to total secrecy [38]. Moreover, men in at-risk families have lower uptake of *BRCA* testing [39,40] and express greater difficulty divulging *BRCA* test results with at-risk relatives [35,39]. The sub-codes we identified within the template theme of ‘gender scripting’ align with observed gender differences in communication and the spectrum of familial response [35,38,39]. Importantly, greater satisfaction with BRCA testing decision (and less regret) is a predictor of open communication in families [18]. Healthcare professionals report barriers to assisting families in effectively communicating risks. Barriers include family lack of understanding about the importance of sharing genetic information with at-risk relatives, family concerns about relatives’ emotional response and complex family dynamics [41]. Indeed, our qualitative interviews of *BRCA*+ women revealed sub-codes (‘harmful negligence’, ‘intra-family ignorance’, ‘paternal guilt’, ‘male stoicism’, ‘active withdrawal of support’) that echo the barriers perceived by healthcare professionals. We and others have proposed guilt and silence as significant roadblocks to open family communication around *BRCA* status [15,16,38,42]. However, families that are able to overcome such reactions and incorporate positive *BRCA* status into their family narrative (reflected in sub-codes of ‘fatherly protection’ and ‘female camaraderie’) can solidify and strengthen the family identity [35]. Thus, theory-based interventions [34] (i.e., drawing on the Theory of Planned Behavior) that target the individual and family may help reframe the threat posed by *BRCA* and support families into achieving a new level of family coping and adaptation.

Sub-codes from the template theme ‘medical biases’ (i.e., ‘medical misconceptions’, ‘medical minimizing’) were frustrating for patients and eroded patient confidence in the healthcare system. Such observations echo findings from previous studies demonstrating that healthcare provider biases contribute to delays in diagnosis and treatment for women with paternally inherited pathogenic *BRCA* variants [8,9,14,43,44]. Lack of training/competencies in genetic healthcare and implicit bias have previously been noted to contribute to poor outcomes [44,45,46]. Importantly, there is increasing attention to the pivotal role healthcare providers play in facilitating intrafamilial communication and subsequent cascade screening [15,47,48,49]. Accordingly, educating clinicians and implementing the recently updated U.S. Preventive Service Task Force recommendations guidelines on risk assessment, genetic counseling and genetic testing for *BRCA*-related cancer is a critical step in rebuilding trust [4]. Health system leadership should acknowledge trust as foundational to care delivery by establishing standards, providing training and measuring trust to ensure accountability [50]. Further, adopting more person-centered approaches to care is an essential component to fostering cascade screening. Shared decision-making helps foster high quality health decisions that are both informed and aligned with patient values and preferences [50,51]. High quality decisions are associated with greater satisfaction and less regret. Thus, more person-centered approaches to care may have indirect positive effects on family communication, thereby facilitating cascade screening [5,18].

Mapping findings to the TPB identified variables to tailor interventions aimed at enhancing family communication of risk and promoting cascade screening. Approaches (Table 5) would aim to reframe traditional male gender scripts away from ‘male stoicism’ to a more adaptive “protector” role wherein being a good man/father is about protecting the family and communicating risk. Men may be reluctant to discuss health and family history, yet for many, sports appears to be an easy and accessible topic of male discussion. Each October since 2009, the National Football League has partnered with the American Cancer Society for the “Crucial Catch” campaign to raise awareness of breast cancer screening, particularly for women over 40 years of age [52]. Changing culture (and overcoming traditional societal gender roles) is a massive undertaking, yet change on an individual level may seem more attainable. In light of findings from the current study and others, perhaps a more successful approach for enhancing cancer screening would be to flip the script. In other words, use the “Crucial Catch” campaign to nudge men to consider their family history of cancer (both maternal and paternal) and share that information with at-risk relatives; thereby embracing the traditional western male role as a provider and protector. Envisioned interventions at the family level could take the form of similar counseling approaches aimed at empowering individuals to assume a role of the family “BRCA informant” to raise family awareness and underscore the importance of cascade screening for early detection, aggressive surveillance and risk-reducing interventions (i.e., hormonal therapies and/or surgery) [53]. The primary focus at the health system level aims to improve genomic healthcare competencies and remove implicit biases by adopting the recently updated U.S. Preventive Service Task Force recommendations for risk assessment, genetic counseling and genetic testing for *BRCA*-related cancer [4]. Such a systems approach (i.e., individual, family, health systems) may be useful as multi-component interventions may be more effective than simply targeting one aspect (i.e., individual, family or institution).

This study has several limitations. First, it is worthwhile to note that we interviewed *BRCA*+ women and observe comments about parental/family behavior though the eyes of the daughter; we did not interview mothers and fathers directly. Second, we cannot assure that findings (particularly regarding gender roles/scripts) are globally transferable due to cultural differences. However, international studies report results that mirror our findings, underscoring a strong desire among most men to keep genetic information private [18,54]. Second, our sample was rather homogeneous. Studies including more diverse patients are needed for generalizability. Third, our recruitment strategy (i.e., online) could have introduced selection bias, as internet access is not universal. Patients frequenting patient support organizations cannot be assumed to be representative of the broader patient population. Similarly, in-depth interview data may not fully represent the entire spectrum of patient experiences. However, our sample size (*n* = 97) is very robust for qualitative inquiry and we can be confident that data saturation was reached. The sample size combined with the iterative approach to data analysis help support the validity of our findings.

## 4. Materials and Methods

The Boston College IRB approved this qualitative research study (protocol #16.109.01) and all participants provided opt-in electronic informed consent prior to participation. Findings are reported according to STrengthening the Reporting of OBservational studies in Epidemiology (STROBE).

### 4.1. Participants and Procedures

A purposive sample was recruited for this study. Women who had tested positive for pathogenic *BRCA* variants were recruited via social media (i.e., Facebook, Twitter) and several online patient-oriented breast cancer sites (Bright Pink, Facing Our Risk of Cancer Empowered [FORCE], National breast Cancer Coalition) (dates: January 2013–September 2016) [55]. Following informed consent, participants (*n* = 97) provided information on sociodemographics, family and personal medical history including genetic counseling and genetic testing experiences. A single study investigator (SHB) conducted all 97 interviews. In-depth telephone interviews (1–1.5 h.) were used to capture women’s experiences being BRCA+. The investigator started each interview by prompting each participant “to share your *BRCA* story” and listened deeply. Additional questions elicited personal and family medical history—including experiences with genetic counseling, genetic testing and probing family communication patterns. Interviews were audio-recorded and transcribed verbatim. Field notes were recorded for each interview. All participants were given the option to review their transcribed interview to edit and clarify responses. No participants chose to review their transcript.

### 4.2. Analysis

Demographic and health history characteristics were compared between groups according to parent of origin (i.e., maternal vs paternal inheritance) using Chi-squared or Fisher’s exact tests as appropriate. To compare age at genetic testing, maternal and paternal inheritance groups were compared according to age range at time of genetic testing (14, 18–25, 26–30, 31–35, 36–40, 41–50, 51–60, and 61–70 years old). Interview transcripts were analyzed using template analysis [56,57,58]. In brief, this approach to qualitative analysis uses a priori coding template (termed “themes”) from the research literature. For this study, template themes were drawn from a comprehensive review of existing literature related to hereditary breast and ovarian cancer, and more specifically, parent of origin (i.e., maternal vs. paternal) inheritance of a pathogenic *BRCA* variant. A particular strength of template analysis is that it follows a structured top-down approach meaning that the existing literature guides the analysis (i.e., template themes for analyzing the qualitative data). A review of the literature published over the past 10-years identified three template themes (gender scripting, family dynamics, and medical bias). Template analysis methodology allows for the modification of the initial template themes with the addition of sub-themes and dimensions during a grounded theory analysis of qualitative data. A grounded theory analysis consists of reading each interview and doing line-by-line coding. A detailed interview memo is created that includes codes with respective line numbers. Two investigators (B.F., S.R.) independently coded the qualitative data and created interview memos. During the analysis process, emergent analytical themes are identified and tested against subsequent interviews. Three investigators (S.H.-B., B.F., S.R.) discussed emergent themes arising from the iterative coding of interview transcripts. All investigators (A.A.D., S.H.-B., B.F., S.R.) agreed to the final coding structure. This iterative coding process allows for a set of analytical categories to emerge as factors clarifying our understanding of the lived experiences of women harboring pathogenic *BRCA* variants. Using a grounded theory approach [59], we derived sub-codes and contributing dimensions corresponding to the a priori template themes. Through the iterative process, data were grouped into sub-codes (representing new analytical ideas) and dimensions (providing additional context and nuance) of the sub-codes that were deductively added to the initial template themes.

### 4.3. Theoretical Framework

We use the Theory of Planned Behavior (TPB) [24] as a guiding theoretical framework for interpreting qualitative findings (i.e., sub-codes, dimensions) around family communication preceding subsequent cascade screening. The central premise of the TPB is that intention precedes action. Intentions may be shaped by attitudes (perceptions of good/bad), subjective norms (expectations of family, friends or healthcare professionals) and perceived control (perceived agency and view that the action is up to the individual). The TPB also posits that attitudes, subjective norms and perceived control are influenced by beliefs/values as well as past experiences [24]. Additionally, we employed an ecological perspective to identify targets for improving care at the individual, familial, and healthcare system levels.

## 5. Conclusions

In summary, qualitative interviews with 97 women identify a matrix of parent of origin and gender role influences on family communication of *BRCA* risk influencing cascade screening. Codes related to blocked intrafamilial communication and medical biases contribute to diminished family *BRCA* awareness and communication of risk. Interestingly, normative beliefs related to roles and gender scripting can pose barriers to communication of *BRCA* risk (i.e., male stoicism and paternal guilt) and contribute to daughters harboring paternally-inherited pathogenic *BRCA* variants feeling “blindsided” by cancer. Gender scripts also limit cascade screening efforts, thereby denying at-risk relatives earlier detection and potentially life-saving intervention. Conversely, gendered scripting can also facilitate family communication (i.e., fatherly protection). Based on findings from qualitative interviews, we propose interventions guided by the Theory of Planned Behavior that attend to human factors influencing intrafamilial communication of BRCA risk and subsequent uptake of cascade screening.

## Figures and Tables

**Table 1:** Sociodemographics of interview participants (*n* = 97 *).

Sociodemographics Heading Title	Paternally Inherited Pathogenic *BRCA* *V*ariant	Maternally Inherited Pathogenic *BRCA* *V*ariant
*race*		
white	29 (85%)	55 (92%)
other	5 (15%)	5 (8%)
*age (years)*		
18–30	7 (21%)	14 (23%)
31–40	12 (35%)	27 (44%)
41–50	11 (32%)	13 (21%)
51–60	3 (9%)	7 (11%)
60+	1 (2%)	1 (1%)
*education*		
<college	−	1 (2%)
college	17 (53%)	39 (67%)
advanced degree	15 (47%)	18 (31%)
*socioeconomic status*		
lower-middle class	5 (18%)	2 (4%)
middle class	20 (71%)	46 (84%)
upper class	3 (11%)	7 (12%)

Paternally inherited pathogenic variant (*n* = 35), maternally inherited pathogenic variant (*n* = 62); * not all participants responded to all questions.

**Table 2:** Representative quotes related to the template theme of gender scripting.

Factors Promoting Intrafamilial Communication of Risk	
**Sub-Code**	**Representative Quote**
Fatherly protection	Participant 026 on her father’s motivation for testing: *“[He said] if it wasn’t for you, I probably wouldn’t get tested. I gave you this mutation, I will help you financially as much as I can [...] don’t worry about it.”*
Female camaraderie	Participant 023 on her paternal aunts who had been documenting family history for decades: *“Luckily [my father] has six sisters and the three that survived were very, you know… proactive in making sure… in keeping all of their sisters’ records, and making sure all of the nieces and their daughters knew, and that… you know, we did see a GYN or OBGYN, and they knew our history going along. In my family, we were really lucky that we had a pretty solid documented history due to my aunts. If my father had six brothers instead of six sisters, we would never have known we had this [BRCA+].”*
**Factors Blocking Family Communication of Risk**	
**Sub-Code**	**Representative Quote**
Male stoicism	Participant 002 on her father ’s lack of emotional expression and reluctance to discuss the family *BRCA*+ history: *“He never really talks about it. I guess he’s never really wanted to know… and I get [it], I totally do understand that. I don’t… like, shame people who don’t want to know, because… I get it.”*
Parental guilt	Participant 033 on her father’s guilt: *“He apologized to me all the time.* *‘I’m so sorry for what I did to you.’ And I,* *‘Dad, you didn’t do anything to me.’ He goes,* *‘I… ya know, I wish… I wish… ya know… I should’ve never had children if I had known’ blah, blah, blah. I said,* *‘Are you kidding? Like, I’m so happy that you had me.’ I… I don’t, but he… he had tremendous guilt.”*

**Table 3:** Representative quotes related to the template theme of family dynamics.

Sub-Code	Representative Quote
Harmful negligence	Participant 030 on lack of urgency regarding *BRCA* despite multiple affected family members: *“She [cousin] just acted like, you know, it was like… nothing. There’s just no communication. I mean, families like that don’t get back together again any more at Christmas, so nobody talks.”*
Intra-family negligence	Participant 044 on invisible/unknown family history: *“He [father] has a tremendous amount of guilt… he feels responsible that, we didn’t have this information sooner. I would never want to say it could have been prevented, but… you think about how we didn’t have a good relationship with his family… perhaps if we did… these conversations… more conversations would have taken place and we could’ve looked a little bit more into what that meant for us, you know?”*
Active withdrawal of support	Participant 035 on her father urging her and the family to ‘move on with their lives’ and subsequently withdrawing support for her: *“I think he became a real jerk about it too. He was just like, just move on… move on with your life already. And I think that’s cause he wanted to move on with his life like I think he felt really guilty for giving it to me.”—* She goes on to explain how this resulted in family conflict and her mother subsequently squashing any conversation about *BRCA* to avoid upsetting her husband and sons.

**Table 4:** Representative quotes related to the template theme of ‘medical biases’.

Factors Contributing to Medical Biases	
**Sub-Codes**	**Representative Quotes**
Medical misconceptions	Participant 031 on her gynecologists genetic misconceptions regarding *BRCA*: *“He had always told me that it was only the mom’s side that we needed to be worried about as far as family history… so that’s obviously not true.”*
Medical minimizing	Participant 036 on her experiences not having medical professionals not considering her family history of a male diagnosed with breast cancer: *“Even when I was diagnosed, which was in 2004, no one was concerned about the fact that I had a male relative [diagnosed with breast cancer] in terms of genetics.”*
**Factors Mitigating Biases**	
**Sub-Codes**	**Representative Quotes**
Informed physicians	Participant 022 on her grandfathers’ medical provider being informed: *“So when my grandfather had the breast cancer, his sister was actually struggling with breast and ovarian cancer at the same time…. and his doctor… given that (history], just brought up that… This is sort of a new finding that… since it’s so rare [breast cancer in a male], and since your sister has breast cancer at the same time… maybe you should be tested for your family’s sake, for this mutation. It’s completely free to get tested, and it might be useful information.”*
Trust in healthcare	Participant 017 expressed her full trust in her healthcare providers when they assured her she was doing “enough” through aggressive surveillance: *“When I go into see my… um, breast doctor… you know, I guess about every other time, you know, I say* *‘Are you sure we don’t need to have my breasts removed?’ And he keeps reassuring me, I am telling you, everything we are doing… if you get cancer, we will catch it so early, so early, you are not going to have to… you are not going to die… you are not going to have to be worried about this. It’s going to be so early”*

**Table 5:** Targets for interventions to improve family communication and cascade screening uptake.

Interventions Targeting the Individual	
**Sub-Codes**	**Potential Interventions**
Fatherly protection (+) Male stoicism (−)	• Framing masculine role as the family protector (i.e., being a good father = sharing information to protect the family)
**Interventions Targeting the Family Unit**	
**Sub-Codes**	**Potential Interventions**
Female camaraderie (+)	• Empowering women to be a “*BRCA* informant” for the family to spread knowledge of risk through the family and highlighting the advantages of early detection and intervention • Genetic counseling to support recognition that genes and inheritance are beyond one’s individual control, reframe the focus to recognize that knowledge is powerful for enabling cascade screening, heightened surveillance and early risk-reducing interventions • Use family systems approach to “nudge” family members to rally around affected members
Harmful negligence (−)	
Intra-family ignorance (−)	
Paternal guilt (−)	
Active withdrawal of support (−)	
**Interventions Targeting the Healthcare System**	
**Sub-Codes**	**Potential Interventions**
Medical misconceptions (−)	• Continuing education (primary care providers, gynecologists) on genomic healthcare competencies (i.e., taking a three generation family history and assessing cancer risk from both sides of the family to enable cascade screening) • Uptake and implementation of U.S. Preventive Service Task Force recommendations for Risk Assessment, Genetic Counseling, and Genetic Testing for *BRCA*-Related Cancer
Medical minimizing (−)	
Informed physicians (+)	
